# Non-nucleotide Agonists Triggering P2X7 Receptor Activation and Pore Formation

**DOI:** 10.3389/fphar.2018.00039

**Published:** 2018-02-01

**Authors:** Francesco Di Virgilio, Anna L. Giuliani, Valentina Vultaggio-Poma, Simonetta Falzoni, Alba C. Sarti

**Affiliations:** Department of Morphology, Surgery and Experimental Medicine, University of Ferrara, Ferrara, Italy

**Keywords:** P2X7 receptor, extracellular ATP, inflammation, cathelicidin, polymyxin B

## Abstract

The P2X7 receptor (P2X7R) is a ligand-gated plasma membrane ion channel belonging to the P2X receptor subfamily activated by extracellular nucleotides. General consensus holds that the physiological (and maybe the only) agonist is ATP. However, scattered evidence generated over the last several years suggests that ATP might not be the only agonist, especially at inflammatory sites. Solid data show that NAD^+^ covalently modifies the P2X7R of mouse T lymphocytes, thus lowering the ATP threshold for activation. Other structurally unrelated agents have been reported to activate the P2X7R via a poorly understood mechanism of action: (a) the antibiotic polymyxin B, possibly a positive allosteric P2X7R modulator, (b) the bactericidal peptide LL-37, (c) the amyloidogenic β peptide, and (d) serum amyloid A. Some agents, such as Alu-RNA, have been suggested to activate the P2X7R acting on the intracellular N- or C-terminal domains. Mode of P2X7R activation by these non-nucleotide ligands is as yet unknown; however, these observations raise the intriguing question of how these different non-nucleotide ligands may co-operate with ATP at inflammatory or tumor sites. New information obtained from the cloning and characterization of the P2X7R from exotic mammalian species (e.g., giant panda) and data from recent patch-clamp studies are strongly accelerating our understanding of P2X7R mode of operation, and may provide hints to the mechanism of activation of P2X7R by non-nucleotide ligands.

## Introduction

The P2X7 receptor (P2X7R) belongs to the ionotropic P2X receptor subfamily (Burnstock, 2006). It was previously known as P2Z, and when first characterized in rat mast cells, it was named the “ATP^4-^ receptor” (Cockcroft and Gomperts, 1980). Identification of the mast cell receptor with the P2X7R cloned by Surprenant et al. (1996) has been questioned on the basis of the widely different apparent Kds for ATP of these receptors, but the functional features are very similar and therefore suggestive that the “ATP^4-^ receptors” and the P2X7R are one and the same molecule. Following experiments have shown that human mast cells express P2X7R-like non-desensitizing currents (Wareham et al., 2009). P2X7R expression and function was also documented in mouse mast cells (Kurashima et al., 2012). Reason for the very high affinity for ATP of the rat mast cell ATP^4-^ receptor is not obvious. For sure, in the original experiments by Bastien Gomperts the ATP Kd was measured in the absence of divalent cations (Ca^2+^ and Mg^2+^), thus it refers to the fully dissociated nucleotide species, and it is well known that threshold for P2X7R activation is lower in the absence of divalent cations. It is also worth mentioning that to our knowledge no analysis of P2X7R expression and function was ever repeated in the same experimental model used by Gomperts, i.e., mast cells obtained by peritoneal lavage of rats pre-immunized with ovalbumin or with antigens from the helminth parasite *Nippostrongylus brasiliensis* (Cockcroft and Gomperts, 1979, 1980). It is therefore possible that P2X7R affinity, and therefore ATP threshold for P2X7R activation, in sensitized mast cells is much higher than in unprimed cells, as shown by the reported up-regulation of this receptor in bronchial alveolar lavage (BAL) macrophages and eosinophils from asthmatic patients (Muller et al., 2011). Eosinophils from asthmatic patients are also more prone to release reactive oxygen species when challenged with the selective P2X7R agonist benzoyl ATP. There is evidence that during infection and inflammation P2X7R affinity is modulated by agents acting on the extracellular domain or at as yet unidentified intracellular residues (Rissiek et al., 2015; Yang et al., 2015). This raises the issue of whether shift in P2X7R activity may naturally occur under pathophysiological conditions, thus permitting P2X7R activation at much lower ATP concentrations. The low affinity of the P2X7R for ATP has been a contentious issue ever since the functional and pharmacological identification and cloning, to the point that its pathophysiological relevance was questioned. On the other hand, it has been argued that the low affinity for ATP is indeed a safe-guard mechanism because it prevents its unwanted stimulation: P2X7R activation in an improper situation may trigger release of potentially injurious inflammatory mediators (e.g., reactive oxygen species, ROS, or IL-1β), or may even precipitate cytotoxicity. In fact, while the ATP concentration in the healthy interstitial tissue is extremely low, i.e., in the nanomolar range, at inflammatory sites it can be as high as a few hundred micromoles/liter (Morciano et al., 2017). Therefore, it is assumed that under physiological conditions the P2X7R should be mostly silent. However, even at the ATP-rich inflammatory sites, with an *in vitro* Kd for ATP of about 0.5–1.0 mM, probability for the P2X7R to be activated is very low. Thus, asking if other agonists besides ATP are active at the P2X7R, or whether physiological positive allosteric modulators may help to lower the activation threshold is not unjustified. On the other hand, given the large repertoire of nucleotide receptors with widely different affinity expressed by most cells, it is likely that even at tumor and inflammatory sites a variable response is generated in the presence of ATP concentrations that may range from the high nano to the low micromolar level.

## Genetics of the P2X7R

The human *P2RX7* gene is located on chromosome 12q24.31, in the proximity of the *P2RX4* gene located at 12.q24.32. Mouse *P2rx7* and *P2rx4* genes are both located on chromosome 5. Close proximity of *P2RX7* and *P2RX4* may suggest an origin by gene duplication (Di Virgilio et al., 2017). Ten, or nine according to a more recent re-evaluation (Sluyter, 2017), splice variants of the human and four of the mouse P2X7 subunit are known. The canonical full-length variant, whether human or mouse, is named P2X7A, while the most common human splice variant, referred to as P2X7B, is a truncated form lacking the last 249 COOH terminal amino acids (aa) and incorporating 18 extra aa after residue 346 (Cheewatrakoolpong et al., 2005). Receptor made by P2X7B assembly retains channel functions but lacks ability to generate the non-selective, large conductance pore considered to be the functional signature of P2X7R. P2X7A and P2X7B may co-assemble on the plasma membrane generating a heterotrimeric receptor with distinct functional properties (Adinolfi et al., 2010). Thus, P2X7B might be considered an “endogenous” modulator of P2X7R activity. Two mouse variants, the natural P2X7k variant, and an artificial hybrid variant occurring in the P2X7-KO mouse originated at Pfizer, have attracted interest because they may escape inactivation in the commonly used P2X7-KO mice (the Pfizer and the Glaxo mice) (Kaczmarek-Hajek et al., 2012). Whether they are also present in a third P2X7-KO mouse described in the literature is not known (Basso et al., 2009). Several loss- or gain-of-function single-nucleotide polymorphisms (SNPs) have been described in the human P2X7 (Di Virgilio et al., 2017; Sluyter, 2017). Combination of these SNPs generates complex haplotypes that affect P2X7R functions, and make basically impossible to predict P2X7R activity on the basis of the mere identification of one SNP. Most interesting SNPs are the loss-of-function rs3751143 1513A>C SNP, which causes replacement of glutamate 496 with alanine (E496A), and the gain-of-function rs208294 489C>T, that causes replacement of histidine 155 with tyrosine (H155Y) (Gu et al., 2001; Cabrini et al., 2005). Additional polymorphisms described in the P2X7 subunit and variably associated to disease susceptibility are: (a) rs17525809, causing replacement of a valine with an alanine (V76A), (b) rs28360447, causing replacement of a glycine with an arginine (G150R), (c) rs7958311, causing replacement of an arginine with an histidine (R270H), (d) rs28360457, that causes replacement of an arginine with glutamine (R307Q), (e) rs1718119, that causes replacement of an alanine with a threonine (A348T), (f) rs2230911, causing replacement of a threonine with a serine (T357S), (g) rs2230912, causing replacement of a glutamine with an arginine (Q460R), (h) rs2230913, causing replacement of a histidine with a glutamine (H521Q), and (i) rs1653624, causing replacement of an isoleucine with an asparagine (I568N) (Wiley et al., 2011; **Table 1**).

**Table 1:** Most common human P2X7 single-nucleotide polymorphisms.

dbSNP ID	Amino acid substitution	Effect on P2X7 function
rsl7525809	V76A	Loss
rs28360447	G150R	Loss
rs208294	H155Y	Gain
rs7958311	R270H	Loss
rs28360457	R307Q	Loss
rs1718119	A348T	Gain
rs2230911	T357S	Loss
rs2230912	Q460R	Loss
rs3751143	E496A	Loss
rs2230913	H521Q	Neutral
rs1653624	I568N	Loss

*From Wiley et al. (2011), Di Virgilio et al. (2017), and Sluyter (2017).*

The most relevant mouse SNP is the P451L missense mutation that changes a proline to a leucine at position 451 (Adriouch et al., 2002). This mutation is constitutively present in C57Black/6, DBA, and C3H strains, while the common laboratory Balb/c strain harbors the P451 allele. The L451 allele reduces P2X7R functions via an as yet undetermined mechanism and has been associated with reduced bone strength and density, and impaired glucose homeostasis (Syberg et al., 2012; Todd et al., 2015).

## Recent Progress In Understanding P2X7R Structure and Function

A mammalian P2X7 subunit deleted of the terminal 240 C-terminal residues (356 aa) from *Ailuropoda melanoleuca* (giant panda) has been crystalized, allowing 3-D reconstruction of the trimeric receptor and identification of the ATP-binding pocket and of allosteric sites (Karasawa and Kawate, 2016). This 3-D reconstruction has allowed identification of the ion pathway through the receptor and of a possible mechanism for allosteric inhibition. Due to the persistent inability to crystallize the full-length P2X7 subunit, i.e., including the long COOH terminal tail, structural modeling has provided no hints as to the molecular mechanism underlying the large and non-selective increase in permeability associated to sustained activation of the P2X7R (the enigmatic P2X7R “macropore”). Nevertheless, analysis of the panda P2X7 subunit, which is 605 aa long, and 85% identical to the human P2X7 subunit, may help decipher the molecular basis of the large permeability increases associated to P2X7R activation (Karasawa and Kawate, 2016; Karasawa et al., 2017).

A solid dogma in this field is that the carboxyl terminal extension of the P2X7 subunit is absolutely needed to support “macropore” formation (Surprenant et al., 1996; Adinolfi et al., 2010), therefore it is assumed that truncated forms of the P2X7 will be of little help to investigate the molecular basis of such a peculiar process. However, it is possible that even a defective P2X7 subunit might provide interesting hints, if properly “interrogated,” and if the answers it offers are placed in the proper context. An example of this is the recent paper by Kawate colleagues in which permeability features of the truncated panda P2X7R were investigated in a model system allowing a controlled modulation of the lipid composition of the bilayer in which the receptor was reconstituted (Karasawa et al., 2017). In this regard, information stemming from the analysis of P2X7R activation by non-nucleotide ligands may well complement those generated by investigation of truncated P2X7Rs, and might provide clues to the molecular mechanism of “macropore” formation.

**FIGURE 1:**
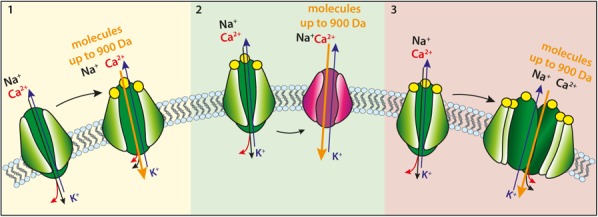
Proposed mechanisms for P2X7R-mediated uptake of molecular solutes up to 900 Da MW. **(1)** Upon sustained or repetitive stimulation with ATP (yellow circles) the P2X7R pore undergoes dilatation to form a high conductance (possibly non-selective) large pore (the macropore); **(2)** activation of the P2X7R recruits in an as yet undefined fashion an accessory molecule (red) that forms the conduit responsible for large aqueous solute uptake; **(3)** sustained or repetitive stimulation of the P2X7R triggers recruitment of additional P2X7 subunits that allow formation of the macropore.

Interest on P2X7R stimulants alternative to nucleotides originated from the finding that some agents (see below) strongly synergize with ATP to stimulate P2X7R-mediated uptake of low MW fluorescent dyes (such as ethidium bromide) (Ferrari et al., 2004), or even induce dye uptake via a P2X7R made by the assembly of carboxyl-terminal truncated P2X7 subunits (P2X7ΔC) in the absence of co-stimulation with ATP (Tomasinsig et al., 2008). This is rather peculiar because homotrimers made by the assembly of subunits lacking the COOH tail (e.g., P2X7B) are generally assumed to be unable to generate the “macropore.” The molecular identity of the pathway underlying “macropore” formation is a contentious issue. Two main hypothesis have been put forward: (1) the P2X7R itself undergoes dilatation during prolonged stimulation, thus transitioning from a cation-selective channel to a non-selective, large conductance pore (Surprenant et al., 1996); (2) the P2X7R itself is a cation-selective channel, intrinsically unable to mediate uptake of high MW aqueous solutes; however, when overstimulated it may recruit additional and yet to identify accessory protein(s) responsible for “macropore” formation (Pelegrin and Surprenant, 2006; Di Virgilio et al., 2017). A variant of the “dilatation hypothesis” holds that overstimulation of the P2X7R might allow recruitment of additional P2X7 subunits (a shift from the canonical trimeric to a hexameric stoichiometry), thus allowing generation of a larger permeation pathway (Ferrari et al., 2004; Di Virgilio et al., 2017; **Figure 1**).

## The ATP-binding Pocket and the Allosteric Site

Some of the agents showed to potentiate ATP-mediated P2X7R activation, as well as some widely used inhibitors, are thought to be allosteric modulators. The ATP-binding site is contributed by two adjacent subunits. Structural analysis revealed three equivalent ATP-binding sites at the interface of each of the three couples of adjacent subunit contact surfaces (Karasawa and Kawate, 2016). The ATP-binding pocket is unique to P2X receptors and bears no similarity to all other ATP-binding sites known. Seven positively charged aa and two hydrophobic residues line the ATP-binding pocket (Jiang et al., 2000; Di Virgilio et al., 2017). Remarkable is the presence of four lysins (Lys64, Lys66, Lys193, and Lys311) which might explain the exquisite sensitivity to P2X7R inhibition by oxidized ATP, a dialdehyde reagent that forms Schiff bases with unprotonated lysins (Murgia et al., 1993). Lack in the P2X7 subunit of a key residue (Ile 217), which is on the contrary present in the ATP-binding pocket of the zebrafish P2X4, might explain lower ATP sensitivity of the P2X7R and higher potency of BzATP (Jiang et al., 2013). Sequence and structural analysis suggests a restricted access and a limited size of the ATP-binding pocket, which might explain why several hydrophilic agonists of other P2XRs are weakly active or fully inactive at the P2X7R.

**FIGURE 2:**
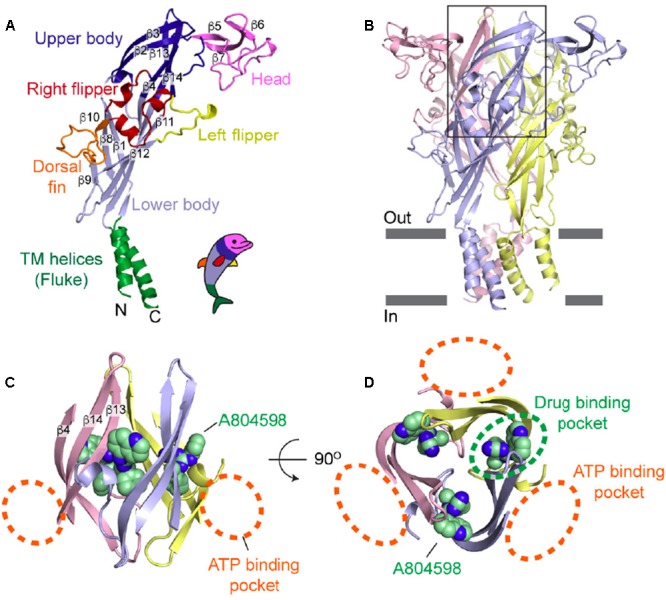
Visualization of the drug-binding pocket of the P2X7R. **(A)** Schematic rendition of the P2X7 subunit in the “dolphin-like” shape. **(B)** Representation of the trimeric panda P2X7R viewed from the side. The black rectangle indicates the approximate location of the area where the ATP-binding pocket and the putative allosteric site are located. **(C)** Side view of the area located in the extracellular domain exhibiting A804598 and ATP-binding pockets (orange dashed lines). **(D)** Top view of the panda P2X7 structure showing the ATP-binding pockets (orange dashed lines) and the drug-binding pocket (green dashed line). Reprinted from Karasawa and Kawate (2016) with permission.

An allosteric-binding site in a pocket made by two adjacent subunits, close and juxtaposed to the ATP-binding site, was identified (**Figure 2**). Occupancy of this site prevents conformational changes associated to P2X7R activation and therefore might hinder movements of P2X7R subunits necessary to allow opening of the ion-conducting pathway (Karasawa and Kawate, 2016). Such an allosteric-binding pocket is absent in P2X4Rs and P2X3Rs, thus highlighting the possibility to develop receptor-selective drugs. Very interestingly, the allosteric site narrows upon ATP binding, thus preventing binding and effect of the allosteric inhibitors. This observation may have important translational implications since it may indicate that in the presence of high concentrations of extracellular ATP this allosteric site will not be accessible, and therefore allosteric P2X7R inhibitors will not be efficacious. This might be a serious inconvenient for the treatment of chronic inflammatory diseases as accumulating evidence shows that ATP may reach a concentration of several hundred micromoles/liter at inflammatory sites (Di Virgilio et al., 2017). Thus, albeit the action of competitive antagonists also depends on the ambient ATP concentration, in the end high affinity competitive antagonists could show higher efficacy than negative allosteric modulators in conditions of high extracellular concentrations of ATP, and might be better suited for the development of P2X7R blockers with higher therapeutic efficacy. Finally, structural analysis did not address the site and possible mechanism of action of agents suggested to be “positive” allosteric modulators, e.g., the antibiotic polymyxin B (Ferrari et al., 2004).

## Modulation Of P2X7R Permeability

Permeability features and low ATP affinity of the P2X7R were a puzzle from the very beginning, to the point that many wondered whether this receptor had any physiological relevance. Nowadays the pathophysiological role of the P2X7R is well established, especially in the context of the immune response. Even the high threshold for activation is not anymore seen as an insurmountable obstacle, since there is now ample evidence that the extracellular ATP concentration can reach hundreds of micromole/liter at inflammatory and tumor sites. However, it has been often wondered whether ATP was indeed the only physiological agonist at the P2X7R, and whether other factors released in the context of pathophysiological responses might synergize with ATP, or even replace it. Or alternatively, the plasma membrane microenvironment might modulate permeability through the P2X7R.

Targeting of the P2X7R to lipid rafts has been shown in several cell systems (Garcia-Marcos et al., 2006a,b; Gonnord et al., 2009; Weinhold et al., 2010), a finding coherent with palmitoylation of several cysteine residues in the COOH tail (Gonnord et al., 2009). Palmitoylation is a reversible post-translational modification assumed to be a plasma membrane localization signal. Covalent modification of protein domains increases the overall hydrophobicity and enhances membrane–protein interaction. For P2X1, P2X2, and P2X4 receptors, reduction of the cholesterol plasma membrane content with methyl-β-cyclodextrin caused inhibition of channel activation (Murrell-Lagnado, 2017), while P2X7R activity was strongly potentiated (Robinson et al., 2014). Kawate colleagues investigated in detail the effect of membrane lipids on P2X7R function, confirming that cholesterol strongly inhibited the P2X7R, and highlighting a facilitating effect of palmitoyl-oleoyl-glycero-phosphoglycerol and of sphingomyelin (Karasawa et al., 2017). These authors went further to identify the mechanism of action of cholesterol suggesting that changes in membrane fluidity are not involved, while on the contrary cholesterol is likely to interact with the transmembrane domains. The effect of the cholesterol content on P2X7R permeability is striking as reconstitution of a truncated form of the panda P2X7R in cholesterol-free liposomes promotes ATP-stimulated fluorescent dye uptake (Karasawa et al., 2017). These latter findings have an additional important implication: they might help shed light on the identity of the “macropore.” Based on current measurements performed in the presence of charge-carrying cations of different sizes it was postulated that formation of the “macropore” is a late event following P2X7R activation, likely due to dilatation of the early cation-selective channel. However, recent electrophysiology experiments clearly showed that the P2X7R exhibits an immediate permeability to large organic cations (NMDG^+^ or spermidine), and that no channel dilatation occurs even during prolonged (30 min) stimulation with ATP (Harkat et al., 2017; Pippel et al., 2017). These findings rule out one of the mechanisms thought to underlay macropore formation, and tilt the balance toward the alternative mechanism that postulate recruitment of an accessory “pore-forming” molecule. However, all attempts so far carried out to identify this hypothetical P2X7R-associated permeability pathway have failed (Di Virgilio et al., 2017). Data by Kawate’s group now give further impetus to the hypothesis that gating of the P2X7R opens a permeability pathway (the “macropore”) intrinsic to the receptor through which cationic fluorescent dyes such as Yo-Pro may permeate the plasma membrane (Karasawa et al., 2017). Similar data documenting Yo-Pro influx were also reported for another member of the P2X family, the P2X2R, by Harkat et al. (2017). On the contrary, we are still left with the unsolved issue of the permeation of anionic fluorescent dyes such as Lucifer yellow (MW 457) or fura-2-free acid (MW 832). Electrophysiological analysis has repeatedly shown that the P2X7R channel is highly selective for cations; thus, it is unclear how anions might permeate. On the other hand if, as currently thought, the P2X7R macropore has no intrinsic selectivity barrier, both cations and anions should be freely permeant.

In principle, Kawate’s group experiments might also rescue the “pore-dilatation” hypothesis because changing the lipid microenvironment of the P2X7R might modulate permeability in such a way to allow a graded increase in pore size. Thus, inability of patch-clamp analysis to document pore dilatation might simply be due to the specific constraints imposed on one side by the transfected cells (e.g., *Xenopus* oocytes), and on the other by the technique (e.g., isolation of individual plasma membrane patches) that might perturb phospholipid mobility in the vicinity of the P2X7R. However, in absence of an experimental proof of these hypotheses, we must stick to the hard data highlighting a discrepancy between description of P2X7R permeability features provided by electrophysiology and cell biology. Electrophysiology and cell biology evidence might be reconciled by assuming that the “macropore” is a separate entity from the P2X7R, i.e., an accessory molecule recruited upon P2X7R activation. This accessory molecule has been long searched for, and general consensus now points to pannexin-1 as the most likely candidate (Pelegrin and Surprenant, 2006). However, the finding that cells from pannexin-1-deficient mouse exhibit basically normal P2X7R-dependent permeability changes casts doubts on pannexin-1 role (Qu et al., 2011; Alberto et al., 2013).

## Extracellular ATP Might Not Be the Only Agonist

Over the time, other agonists, nucleotides such as NAD, or completely unrelated agents such as cathelicidins, have been suggested to activate the P2X7R. The best documented example of a “non-ATP” agonist case is NAD^+^ in mouse cells. Mouse cells express the plasma membrane enzyme ADP-ribosyltransferase (ARTC2.2) that catalyzes transfer of an ADP-ribose moiety from NAD^+^ to arginine 125, close to the ATP-binding pocket of the P2X7R (Seman et al., 2003). ADP ribosylation (being a covalent modification) causes a long-lasting activation of mouse P2X7R which can be reversed by the NAD^+^-degrading enzyme ecto-NAD^+^-glycohydrolase (CD38). It is not entirely clear whether NAD^+^ is a true P2X7R agonist or whether it lowers the activation threshold for ATP, thus sensitizing the P2X7R to autocrine/paracrine-released ATP. Anyway, since an increased NAD^+^ content has been shown at inflammatory sites (Adriouch et al., 2007), it is likely that NAD^+^ has a role in the pathophysiological mechanism of P2X7R activation. Of the four splice variants expressed in the mouse, the P2X7k subunit preferentially expressed by T lymphocytes is the most sensitive to ADP-ribosylation (Rissiek et al., 2015).

A few non-nucleotide agonists are reported to activate the P2X7R (Di Virgilio et al., 2017). Given the structural constraints set by the ATP-binding pocket, it is likely that these agents either stimulate the P2X7R secondarily to ATP release, or act as positive allosteric effectors. The amyloidogenic β peptides Aβ 1–42 or Aβ 25–35 trigger several P2X7R-associated responses in mouse microglia, such as Ca^2+^ influx and ethidium bromide uptake, cell rounding and swelling, IL-1β release, and cytotoxicity (Sanz et al., 2009). All such responses are abrogated by P2X7R blockade, and are absent in microglia cells isolated from P2X7R-deficient mice. The finding that amyloid β also triggers ATP release from microglia, and that amyloid β-dependent permeabilization of microglia plasma membrane is abrogated by co-incubation in the presence of apyrase, might suggest that P2X7R activation is secondary to ATP release. However, two findings are at odd with this interpretation: firstly, amyloid β-stimulated ATP release is inhibited by pre-treatment with oxidized ATP and absent in microglia from P2X7-deficient mice; secondly, addition of amyloid β strongly potentiates ATP-dependent cytotoxicity (Sanz et al., 2009). Lack of ATP release in the absence of a functional P2X7R might suggest that the P2X7R itself is a pathway for ATP release, or alternatively that amyloid β directly stimulates the P2X7R. Furthermore, potentiation of ATP-dependent cytotoxicity by amyloid β is unlikely due to further ATP release since it occurs in the presence of maximally stimulatory ATP concentrations. It cannot be excluded that amyloid β acts as a positive allosteric modulator that lowers the activation threshold for ATP. The inhibitory effect of apyrase on amyloid β-stimulated P2X7R activation can also be re-interpreted along these lines, since amyloid β would be ineffective in the absence of the physiological ATP ligand. The P2X7R is currently heavily investigated as a druggable target for the treatment of neurological pathologies, Alzheimer’s diseases included, albeit no P2X7R-targeting drugs have yet been taken to clinical trials for central nervous system diseases (Di Virgilio et al., 2009; Bhattacharya and Biber, 2016). Activation of the P2X7R by amyloid β suggests the possible use of P2X7R blockers to treat Alzheimer’s and other neurological diseases.

Besides amyloid β, serum amyloid A (SAA) has also been suggested to directly stimulate the P2X7R (Niemi et al., 2011). However, in these experiments IL-1β release from human macrophages was used as readout, which is a rather indirect measurement of P2X7R activation. Rather surprisingly, IL-1β release was not inhibited by treatment with apyrase, but in fact enhanced. These findings suggest that SAA does not induce ATP release, but rather directly interacts with the P2X7R. However, it is not clear why apyrase treatment increased SAA efficacy.

**FIGURE 3:**
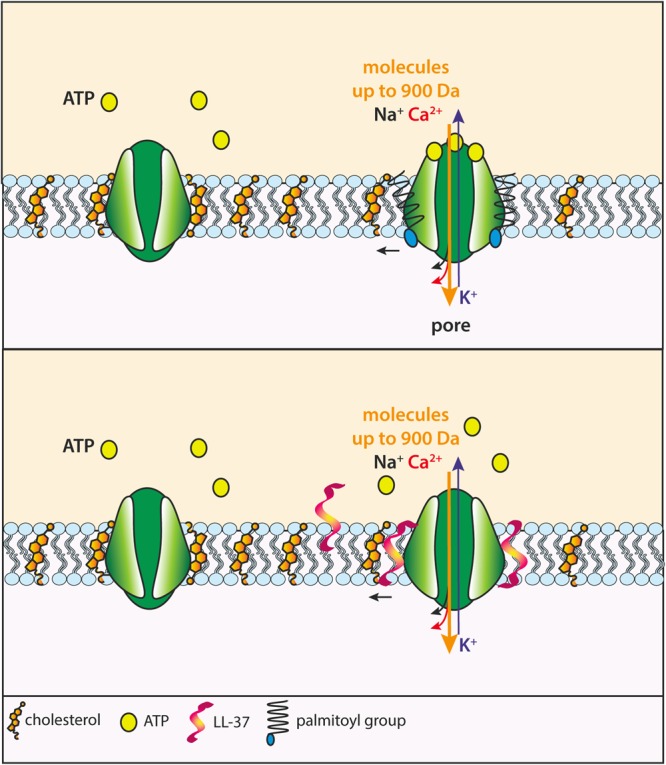
Proposed mechanism of facilitation of macropore formation by the COOH tail of P2X7 subunits and hypothetical mechanism of action of the bactericidal peptide LL-37. The COOH tail of P2X7 subunits harbors several cysteines that can be palmitoylated. Upon ATP binding to P2X7R, palmitoylation increases interaction of the COOH tail with the plasma membrane in the vicinity of the P2X7 TM2, thus relieving the inhibitory effect of cholesterol, thus allowing opening of the macropore **(Upper)**. The LL-37 peptide inserts into the plasma membrane in the vicinity of the P2X7R and repels cholesterol away from the receptor, thus removing its inhibitory action **(Lower)**.

More interesting is the activity of the bactericidal peptide cathelicidin LL-37. Cathelicidins are a family of endogenous antimicrobial peptides found in mammals where they are either constitutively expressed or induced following injury and inflammation (Frohm et al., 1997; Dorschner et al., 2001). LL-37 is the only cathelicidin present in humans where it is stored within granulocytes, lymphocytes, macrophages, and epithelial cells. It was originally shown by Wewers colleagues that LL-37 activates the P2X7R of human monocytes thus causing YO-Pro uptake and IL-1β release (Elssner et al., 2004). LL-37-stimulated effects could be blocked by P2X7R antagonists and, despite LL-37 stimulation induced some ATP release, were independent of ATP release. As a follow up to these studies, we showed that LL-37 triggered Ca^2+^ influx and ethidium bromide uptake in HEK293 cells transfected with the human P2X7R (Tomasinsig et al., 2008). LL-37 stimulation replicated the growth-promoting effect described as a result of low-level, tonic, stimulation of the P2X7R (Adinolfi et al., 2005) as it promoted proliferation of mouse NIH3T3 fibroblasts or P2X7R-transfected HEK293 cells, but not of wild-type, P2X7R-less HEK293 cells (Tomasinsig et al., 2008). Even more intriguing is the effect of LL-37 in HEK293 transfected with the rat P2X7 construct truncated at position 415 (P2X7ΔC). This truncated subunit assembles into a receptor that supports cation fluxes but is unable to form the non-selective macropore (Surprenant et al., 1996). Incubation in the presence of LL-37 allowed formation of the non-selective macropore even in HEK293 cells transfected with the defective P2X7ΔC subunits (**Figure 3**).

LL-37 can be considered an endogenous antibiotic. Another antibiotic of bacterial origin, polymyxin B, derived from the bacterium *Bacillus polymyxa*, also acts as a positive allosteric P2X7R modulator (Ferrari et al., 2004, 2007). Polymyxin B potentiates P2X7R-dependent Ca^2+^ uptake, plasma membrane permeabilization, and cytotoxicity in mouse and human macrophages and in HEK293 and erythroleukemia K562 cells transfected with the P2X7R. Incubation in the presence of polymyxin B also potentiated ATP-stimulated cytotoxicity in leukemic B lymphocytes. Polymyxin B might be a tool for the investigation of the pathway for P2X7R-dependent fluorescent dye uptake. Several years ago we showed that co-incubation in the presence of this antibiotic and ATP induces the appearance of a 440-kDa band in western blots stained with anti-P2X7 antibodies (Ferrari et al., 2004). We interpreted this band as a higher order aggregation form (hexamer?) of the P2X7 subunit. In the same blots we also detected a 220 band, possibly related to the canonical trimer.

Gating of the P2X7R may be triggered by agonists acting on the cytoplasmic side. We reported several years ago that macrophage activation by lipopolysaccharide (LPS), the paradigmatic bacterial endotoxin, involves ATP release and P2X7R activation (Ferrari et al., 1997). Recently, a study by Nunez and coworkers has provided an intracellular mechanism to explain this effect by showing that cytoplasmic LPS lowers the threshold of P2X7R activation, sensitizing this receptor to ambient ATP concentrations, and thus triggering P2X7R-dependent responses (Yang et al., 2015). P2X7R modulation by cytoplasmic agents is also supported by the finding that Alu-RNA accumulation in the cytoplasm can activate P2X7R independently of ATP release (Fowler et al., 2014).

**FIGURE 4:**
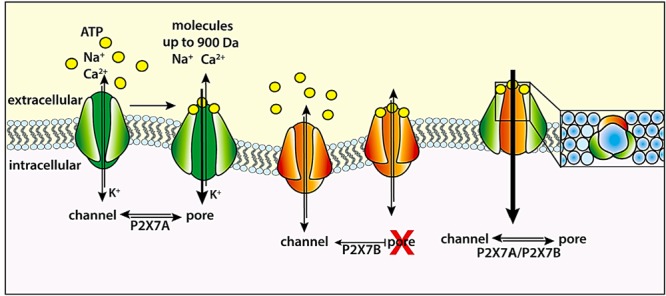
The P2X7B as an endogenous modulator of the P2X7R. The P2X7R formed by full-length P2X7 subunits (green) can function as both an ion channel and a large conductance pore (macropore). The truncated P2X7B (orange) is unable to generate the macropore. The P2X7B can assemble with P2X7A to form a heterotrimeric P2X7A/P2X7B (green and orange) receptor that sustains higher Ca^2+^ influx and ethidium bromide uptake compared to the homotrimeric P2X7A receptor (Adinolfi et al., 2010).

Activity of the P2X7R macropore can be also affected by changing the splice variants expressed. It is well known that 10, or 9 according to some authors (Sluyter, 2017), human P2X7 splice variants are present, P2X7A being the canonical full-length monomer (Di Virgilio et al., 2017). A common variant is the truncated P2X7B isoform. The receptor resulting from P2X7B monomer assembly shows ion channel activity but no macropore function. However, co-expression of P2X7B together with P2X7A leads to formation of a functional P2X7A–P2X7B heterotrimeric receptor that shows enhanced macropore function compared to the homotrimeric P2X7A receptor. The heterotrimeric P2X7A–P2X7B receptor shows higher affinity for ATP or BzATP, and an enhanced capability to support cell energy metabolism and proliferation (**Figure 4**).

## Conclusion

Ever since its molecular cloning and functional characterization it was assumed that the only physiologically relevant agonist of the P2X7R was extracellular ATP. Accruing evidence from various laboratories now shows that other factors may gate this receptor thus revealing an entirely novel and exciting scenario where multiple agents produced during inflammation may converge on this receptor to trigger release of pro-inflammatory factors and even cytotoxic reactions. Furthermore, novel data suggest that permeability through the P2X7R can also be modulated from the inside of the cell, albeit the mechanism involved is utterly unknown. Finally, resolution of the 3-D structure of the full-length receptor, i.e., COOH tail included, will certainly bring novel exciting information on the mechanism underlying P2X7R permeability changes.

## Author Contributions

FDV delineated the outlines, wrote a section, and edited the whole review. ALG wrote a section in the review and contributed to others. VV-P wrote a section of the review. SF wrote a section on the review. ACS wrote a section, contributed to overall writing of the review, and took responsibility for iconography.

## Conflict of Interest Statement

FDV is a member of the Scientific Advisory Board of Biosceptre, Ltd., a UK-based biotech company involved in the development of P2X7R-targeted therapeutics. The other authors declare that the research was conducted in the absence of any commercial or financial relationships that could be construed as a potential conflict of interest.
